# Audiologists’ presbycusis and associated tinnitus counselling practices within the KwaZulu-Natal province

**DOI:** 10.4102/sajcd.v69i1.869

**Published:** 2022-09-08

**Authors:** Kerusha Bhojraj, Vuyelwa Z. Peter

**Affiliations:** 1 Department of Health, Durban, South Africa; 2 Discipline of Audiology, University of KwaZulu-Natal, Durban, South Africa

**Keywords:** presbycusis, tinnitus, counselling, patient-centred care, counselling practices, multi-cultural

## Abstract

**Background:**

Counselling plays an integral part in the management of presbycusis and tinnitus. Counselling can be conducted by implementing patient-centred care (PCC), showing improved patient outcomes; however, it has been poorly implemented in healthcare in South Africa (SA), particularly in the field of audiology for this condition.

**Objectives:**

This study aimed to report on audiologists’ presbycusis and tinnitus counselling practices as guided by the PCC framework within KwaZulu-Natal (KZN).

**Method:**

Quantitative semi-structured online surveys were conducted using purposive sampling of 41 audiologists practising in both the public and private sectors within KZN.

**Results:**

This study revealed that 7.3% (*n* = 3) of participants provided only compensation for hearing loss without counselling, and only 2.4% (*n* = 1) of participants offered group counselling. Apart from this, 7.9% (*n* = 3) reported that tools and resources in counselling were not useful, whilst 12.5% (*n* = 5) reported that counselling was not multiculturally sensitive. Furthermore, a *p* = 0.044 suggests the public sector reported a dire need for improved training in counselling as compared with the private sector.

**Conclusion:**

Audiologists have been providing services within PCC to the best of their capabilities and their environments; however, there is still room to better implement PCC for improved patient outcomes. PCC has not been fully implemented into counselling practices in terms of patient preferences, emotional support, involvement of significant others, integrated care and education.

## Introduction

The Global Burden of Disease (GBD) established that hearing impairment is one of the top five leading causes of disability worldwide (Global Burden of Disease et al., 2017). In 2019, an estimated 1.57 billion people globally presented with a hearing impairment (Global Burden of Disease, 2021). Whilst prevalence data of hearing loss in South Africa are scarce, sub-Saharan Africa was ranked as the third-highest region affected by this disabling hearing impairment, with an estimated prevalence of 4.55% within its population (World Health Organization, 2018). The GBD further predicts an increase in the number of the hearing-impaired population over the years because of an increase in lifespan, as derived from data indicating that 62.1% of those with hearing impairment were older than 50 years (Global Burden of Disease, 2021). Based on the nature of hearing impairment, a possible amelioration is to improve healthcare provision (Global Burden of Disease, 2021), focusing on presbycusis as it is the leading cause of hearing impairment in adults. Presbycusis is an age-related hearing loss, documented as the leading cause of hearing loss within the adult population; it is accompanied by many symptoms, primarily tinnitus, a perception of noise in the ears (Cunningham & Tucci, 2017; Jafari, Kolb, & Mohajerani, 2019; Lee, 2013; Sogebi, Olusoga-Peters, & Oluwapelumi, 2014; Zhang, Yu, & Ruan, 2020). Exploring audiologist practices for presbycusis and tinnitus is therefore crucial for improved services and patient satisfaction, especially in resource-constrained contexts with cultural and linguistic diversity within South Africa.

Presbycusis is a complex phenomenon because of its associated symptoms (Zhang et al., 2020). Research has determined that presbycusis starts to manifest as early as 30 years and can lead to cognitive decline in adults, communication difficulty, isolation and depression, causing psychosocial effects that negatively impact the quality of life (Kim & Yeo, 2015). Similarly, tinnitus that tends to co-occur with presbycusis is a debilitating symptom with similar psychosocial effects and impact on quality of life (Chari & Limb, 2018). Sogebi et al. (2014), supported this claim through research conducted in Nigeria by exploring the symptoms of presbycusis in 69 patients. They found that 79.7% of participants reported tinnitus, thereby indicating that tinnitus is associated with presbycusis and thus suggesting an early onset of presbycusis and high co-existence of tinnitus.

Research indicates that there is currently no medical cure for presbycusis and tinnitus (Swain, Nayak, Ravan, & Sahu, 2016). Current treatment options in the literature include pharmacological drug administration for tinnitus and non-pharmacological forms of treatment for tinnitus and presbycusis (Han, Lee, Kim, Lim, & Shin, 2009). Whilst studies found that patients with tinnitus showed a preference for pharmacological treatments (Swain et al., 2016), a combination of both forms may be identified as the most effective management of presbycusis and tinnitus (British Society of Audiology, 2016; Parham, Lin, Coelho, Sataloff, & Gates, 2013). However, the most common management strategy comprises compensating for the hearing loss (Parham et al., 2013). Whilst this may provide amplification and mask the tinnitus, it does not adequately address the patient’s psychosocial needs (Parham et al., 2013). These differing views emphasise the need for a management approach that can embrace and allow for holistic management to ensure patient benefit, which includes the best of both worlds (British Society of Audiology, 2016; Parham et al., 2013; Swain et al., 2016). Holistic management is therefore suggested to include counselling as a crucial aspect to improve the management of presbycusis and tinnitus (Parham et al., 2013). This goes beyond just compensation for hearing loss, addressing the life-long impact of presbycusis and tinnitus, thus improving the patient’s quality of life and outcomes from services (Parham et al., 2013).

As part of the services offered, counselling is an integral component of the audiologist’s scope of practice to inform and assist patients in adjusting to the psychosocial effects of hearing impairment. It is conducted post-audiological assessment, with differing counselling practices based on the patient’s audiological needs and underlying pathology (American Speech Hearing Association, 2012). These counselling practices as excerpted from the literature utilise counselling methods, counselling efficacy tools and audiological rehabilitative training, ensuring multicultural sensitivity and patient satisfaction (American Speech Hearing Association, 2012). Research on counselling practices within the field of audiology is scarce (Meibos et al., 2017), particularly within developing countries with diverse cultures, languages and needs. Whilst reasons for the paucity of research are unknown, audiologists’ inclination to focus on compensation for hearing impairment rather than on the psychosocial aspects may be an anecdotal attribution. Moreover, the literature supports service provision that is sensitive and inclusive of patient needs (Parham et al., 2013).

Audiologists have advocated that counselling practices should be customised to the patient’s individual needs (Hoare, Gander, Collins, Smith, & Hall, 2012). This requires an approach that focuses on the patient’s individual needs. The patient-centred care (PCC) approach fits such a requirement as it focuses on the patient’s individual needs and encourages active participation in the decision-making process regarding treatment whilst addressing medical needs (Nkrumah & Abekah-Nkrumah, 2019). Within the field of audiology, PCC must be implemented to ensure appropriate counselling, as some sectors of the literature suggest that power dynamics between the audiologist and patient need to be carefully considered during management to improve patient–clinician dynamics and increase shared decision-making (Elwyn et al., 2014). Other sectors of literature proposed that patients may be inclined to take the back seat in decision-making, allowing their clinicians to lead (Schoenfeld et al., 2018). Research has indicated that audiologists present as dominating during the management of patients (Shah, 2020), without allowing patients to fully express themselves or acknowledging their preferences (Shah, 2020). It can be speculated that this is the case with presbycusis and tinnitus counselling. To allow patients to fully express themselves and their preferences during management, the authors advocate audiological counselling practices to be delivered through adopting and implementing PCC (Elwyn et al., 2014).

The PCC framework was first introduced in 1964, based on the eight Picker principles (Picker Institute Europe, 2020), namely (1) respect for patients’ values, (2) preferences and expressed needs, (3) coordination and integration of care, (4) information sharing, (5) comfort and support for the patient and significant others, (6) involvement of significant others, (7) continuity and (8) access to care (Picker Institute Europe, 2020). Patient-centred care proposes that the patient’s individual psychological needs should be the point of focus when providing health services (Epstein & Street, 2011). This framework advocates for the involvement and inclusion of the patient’s significant others and the patient’s active participation in shared decision-making during the management process (Picker Institute Europe, 2020). DiLollo and Neimeyer (2020) suggested that patient-focused healthcare services improve patient satisfaction. Therefore, PCC serves as an ideal influence in audiology counselling practices. Patient-centred care advocates that the patients’ beliefs, preferences and perspectives serve as an integral part of healthcare delivery and may be utilised to improve counselling practice guidelines (Montori, Brito, & Murad, 2013). These are critical aspects in a culturally and linguistically diverse context such as South Africa.

Patient-centred care has gained much popularity in the healthcare system, both locally and internationally. However, research on audiologists’ presbycusis and tinnitus counselling practices require further study to investigate implementation of PCC. Although the benefits of PCC are explored in literature, research on the implementation during counselling practice in audiology is limited. Boisvert et al. (2017) found that although there is an awareness of PCC within the field of audiology, it is not effectively being implemented in practice in sub-Saharan Africa. This poor implementation is attributed to strained resources, poor interpersonal patient–healthcare provider interaction, and lack of focus on psychosocial impacts of illnesses in healthcare training (De Man et al., 2016). Therefore, research on PCC during audiology counselling practices for presbycusis and tinnitus counselling is crucial in closing the knowledge gap and aiding in conceptualising PCC implementation in a developing context. Hence, this study aims to explore presbycusis and associated tinnitus counselling practices in this context by answering the following research question: what are the presbycusis and tinnitus counselling practices offered by audiologists within KZN?

## Methodology

### Aim and objectives

This study aims to report on audiologists’ presbycusis and associated tinnitus counselling practices, guided by the PCC framework within KZN.

### Study design and context

This non-experimental descriptive study was conducted with self-administered online surveys developed by the researcher to best explore audiologists’ presbycusis and tinnitus counselling practices. The online self-administered survey had been adopted from the Picker principles in PCC (Picker Institute Europe, 2020), serving as the following fundamental components of counselling practices: information, education, coordinate-integrated care, respect for patient preference, access to care, emotional support and involvement of significant others. This allowed for information to be gathered on current counselling practice, skills and challenges audiologists are faced with whilst conducting presbycusis and tinnitus counselling. A descriptive design helps to collect information based on a specific field; it can be used to gather information on matters related to current practices or to determine practices amongst healthcare professionals (Brink, Van der Walt, & Van Rensburg, 2012). This enabled the authors to obtain information on current counselling practices conducted by audiologists. Furthermore, the online survey efficiently covered a wide geographic sample, whilst ensuring ethical advantages (Fox, Hunn, & Mathers, 2009). It allowed for efficient data collection around KwaZulu-Natal during the worldwide pandemic of COVID-19 and provided a safer method of obtaining data.

### Data collection tool

This study used self-administered online surveys with open-ended questions to enable quantitative data to be collected over a wide geographical area (Brink et al., 2012). The survey was guided by the Picker principles (Picker Institute Europe, 2020). In addition, the researcher adopted certain sections from the tool aligned with this study (Picker Institute Europe, 2020). The survey was approximately 20–30 min long and efficiently covered a broad geographic sample (Fox et al., 2009). Questions included Likert scales, close-ended questions and an option to elaborate on questions. In addition, the tool included the following sections: biographical data, counselling methods, counselling efficacy and tools, audiological rehabilitative training, multicultural sensitivity and patient satisfaction.

### Study site, population and sampling technique

Purposive sampling was conducted that ensured participants were representative of the phenomena under research (Brink et al., 2012). The survey was conducted on 41 audiologists practising within the 11 districts of KZN. This ensured that the sample is heterogeneous and representative of the general population (Leedy & Ormrod, 2013). The inclusion criteria were based on audiologists registered with the Health Professions Council of South Africa, practising within KZN in both public or private sectors that provide presbycusis and tinnitus counselling to adults.

### Data collection procedure

Once ethical clearance was obtained, the relevant gatekeepers from the public and private sectors were contacted to allow the progress of the study. These gatekeepers included the provincial Department of Health, owners of private practices, hospital management and professional associations’ body chairpersons. Gatekeepers serve as mediators for accessing study participants to obtain data required for research (Andoh-Arthur, 2019). Once gatekeeper permission was obtained, the public Speech Language Therapists and Audiologists Forum and private regulatory boards such as the South African Speech-Language and Hearing Association and South African Audiology Association (SAAA) were contacted via email to obtain permission to progress with the study and obtain participant details. Representatives from SAAA shared the invitation to participate in the study request via email to the database of private audiologists in KZN. Audiologists who wanted to participate then read the invitation letter and information and consent form. The information and consent form with the link to the self-administered survey was then sent via email to all audiologists within the private sector in KZN. Once gatekeeper permission was received from the public sector, the information and consent form and the link to the self-administered survey were shared with the audiologists in KZN via email addresses obtained from the Speech Language Therapists and Audiologists Forum. The information and consent form was sent to all participating audiologists in both public and private sectors to inform them of the study and that clicking on the link to the online survey meant that they consented to participating in the study. The information and consent form and survey link were therefore circulated to 53 private audiologists via SAAA’s contact database and 83 audiologists within the public sector. Three weeks later, the researcher sent out a gentle reminder via e-mail for participants to complete the survey. Three weeks after the first reminder, the researcher sent a second gentle reminder to all participants via email to complete the survey. A total of two reminders were sent, encouraging participants to complete the online survey to improve the response rate. The participants were given a 2-month timeframe to complete the survey before closing the responses.

### Data analysis

Data collected were then coded onto a Microsoft Excel spreadsheet before using the Statistical Package for Social Sciences version 27 to capture and analyse data. Descriptive statistics (mean, standard deviations and range) and inferential statistics, Pearson’s chi-square and Fisher’s test were used to analyse the data. Chi-square testing was selected because of the robustness regarding data distribution, detailed information and convenience of computing data (McHugh, 2013). It was used to compare variables for statistical significance. A significance level of *p* < 0.05 was set for data analysis that indicated a 5% risk of concluding an association between the variables exists when there is no actual association (McHugh, 2013). A statistician was consulted on the selection of statistical tests that best suited the study, taking into consideration sample size and the nature of research, to ensure the increased quality of data analysis.

### Reliability and validity

A pilot study was conducted prior to ensure reliability and validity of data collected during the main study, which provided adequacy, ordering and comprehensiveness of research tools used (Regmi, Waithaka, Paudyal, Simkhada, & Van Teijlingen, 2017). The pilot study identified possible challenges or problems that would be encountered during the study. The study was then altered and modified, ensuring that results remained valid and reliable (Leedy & Ormrod, 2013). The pilot study provided testing of data collection recruitment methods and determining the proficiency of the data collection instrument. In addition, it aided in identifying any modifications that were made. The pilot study was conducted with two audiologists within the KZN region. Two audiologists completed the online surveys; these audiologists were practising within the public sector of KZN, both being female, one with 31–40 years of experience and the other with between 41 and 50 years of experience. All participants included in the pilot study were not included in the main study. However, the pilot study followed the same data collection methods stipulated in the main study’s methodology section, following the same inclusion and exclusion criteria. Furthermore, a statistician was consulted to ensure the reliability of statistical tests and data.

### Ethical considerations

Ethical clearance was obtained from the Humanities and Social Sciences Ethics Committee of the University of KwaZulu-Natal (UKZN) with the reference number HSSREC/00002114/2020. The study adhered to the Declaration of Helsinki code of conduct for ethical research (Goodyear, Krleza-Jeric, & Lemmens, 2007). The Declaration of Helsinki is a code of ethical principles used during human research in which the researcher ensures that the well-being and respect of participants takes precedence throughout the study process, mainly through informed consent, autonomy and anonymity (Goodyear et al., 2007).

## Results

The survey link was sent out to 136 audiologists in KZN from both public and private sectors; from these audiologists, with guidance from a statistician, a sample of 50 audiologists who met the required criteria was created. This was concluded to be an adequate size for statistical analysis from a population of an estimated 136 audiologists. From these audiologists, taking into account the sample size, 41 (82%) responses were obtained from audiologists with differing demographics as shown in Table 1.

**TABLE 1:** Description of participants (*n* = 41), in relation to years of experience.

Category	Subcategory	*n* = 41	%	Years of experience (*p*)
Gender	Female	33	80.5	0.182
	Male	8	19.5	
Age	< 30	26	63.4	0.000
	31–40	9	22.0	
	41–50	0	0	
	51–60	6	14.6	
Sector of employment	Private sector	12	30.0	0.003
	Public sector	28	70.0	

Table 1 shows that participants were mostly female (80.5%) and showed no significant differences with years of experience. Most participants fell within the age range of 30 and below, which accounted for 63.4% (*n* = 26), and a significant relationship between age and years of experience amongst participants was observed; this was because of the younger age of participants showing fewer years of experience. Furthermore, 30% (*n* = 12) participants were practising in the private sector and 70% (*n* = 28) were in the public sector, and a significant relationship was observed between sector of employment and years of experience (*p* = 0.003). This is further detailed in Table 2.

**TABLE 2:** Participants’ years of experience in relation to sector of employment.

Category	Sub-category	*n* = 41	%	Sector of employment (*p*)
Years of experience post-graduation	< 1	9	22.0	0.003
	1–4	5	12.2	
	5–10	21	51.2	
	11–20	3	7.3	
	21–30	0	0	
	> 30	3	7.3	

Whilst one participant did not state his or her sector of employment, Table 2 indicates that the majority of *n* = 40 participants (51.2%) attained between 5 and 10 years of experience and consisted mostly of participants from the public sector. Furthermore, participants with more than 30 years of experience, *n* = 3, are exclusively practicing in the private sector.

Results were further presented in a suite of counselling practices excerpted from literature as (1) counselling methods, (2) counselling efficacy and tools, (3) audiological rehabilitative training, (4) multicultural sensitivity and (5) patient satisfaction. These are derived from the underpinning theoretical framework that guides the study. Furthermore, participants’ sectors of employment and years of experience were analysed as variables of interest and were closely inspected when significant relationships were established.

### Counselling methods

Counselling methods used by audiologists include management through hearing assistive devices, counselling conducted through informational or adjustment counselling and methods of delivery such as individual, group or family counselling (American Speech Hearing Association, 2012).

**FIGURE 1:**
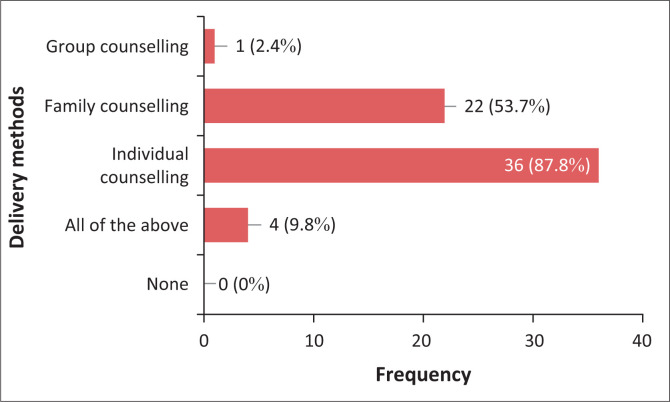
Delivery methods used during counselling.

Amongst 41 audiologists, 34 (82.9%) managed presbycusis and tinnitus through compensation for the hearing loss with an assistive device and provided counselling services, whilst four (9.8%) participants were provided only counselling and three (7.3%) provided only compensation for hearing loss with an assistive device, without counselling. There was no significance with years of practice (*p* = 0.072) or sector of employment (*p* = 0.968) impacting a management course. Apart from management, counselling was conducted through informational and adjustment counselling. The majority of audiologists 75.6% (*n* = 31) offered both informational and adjustment counselling whilst 24.4% (*n* = 10) offered only informational counselling during presbycusis and tinnitus counselling. There was no significant relationship between informational and adjustment counselling and years of practice (*p* = 0.0718) or sector of employment (*p* = 0.426). The study further focused on delivery methods used during counselling as depicted in Figure 1.

Figure 1 revealed that 87.8% (*n* = 36) of participants offered counselling through individual counselling, whilst 53.7% (*n* = 22) offered family counselling, 9.8% (*n* = 4) offered all methods of delivery and only 2.4% (*n* = 1) offered group counselling; all participants offering group counselling practised within the public sector. There was no significant relationship between delivery methods and years of experience (*p* = 0.343) or sector of development (*p* = 0.322). A total of 24 participants (*n* = 24) reported on reasons for the implementation of chosen methods of delivering counselling. The study found that 8.33% of participants (*n* = 2) reported that individual counselling was attributed to the COVID-19 pandemic preventing group counselling. Furthermore, 20.8% (*n* = 5) stated that individual counselling allowed discretion and additional time spent focused on the patient to accommodate specific needs. However, 4.16% (*n* = 1) reported that the private sector prefers individual counselling over group and family counselling, possibly because of telehealth only allowing for individual counselling.

### Counselling efficacy and tools

Within this study, the efficacy of counselling refers to the benefits or helpfulness of counselling practices (Parham et al., 2013). To assure the efficacy of presbycusis and tinnitus counselling practices, audiologists must ensure the use of appropriate measurement scales (British Society of Audiology, 2016), which for this study are considered as tools or resources. This aspect of counselling practices therefore focuses on the tools and resources used by audiologists. Participants reported use of the following tools and resources when counselling patients with presbycusis and tinnitus: Abbreviated Profile of Hearing Aid Benefit, Tinnitus Handicap Inventory (THI), Self-Assessment of Communication Hearing Handicap Scale for the Elderly (SCHHSE), questionnaires, models and pamphlets. From 38 responses, 92.1% (*n* = 35) found these tools useful during practice whilst 7.9% (*n* = 3) reported it not useful; although participants were allowed to elaborate on the tools’ usefulness or otherwise, there were no reasons stipulated for tools being not useful during counselling practices. Significant relationships were found between the efficacy of tools used during counselling practices, as depicted in Table 3.

**TABLE 3:** Relationship between the efficacy of tools and resources to years of experience and sector of employment.

Category	Years of experience (*p*)	Sector of employment (*p*)
Efficacy of tools and resources	0.049	0.094
Multicultural sensitivity of tools	0.643	0.805
Understanding of tools and resources	0.383	0.048

There is a significant relationship between the efficacy of tools and resources and participants’ years of experience, resulting in a *p* = 0.049; 75% of responses disagreed on current tools and resources being of efficacy during counselling practices, and these participants fell within the group with 5–10 years of experience. There was no significant relationship between the efficacy of tools and resources and sectors of employment. Whilst there was no relationship between the multicultural sensitivity of tools and resources and years of experience (*p* = 0.643) and sector of employment (*p* = 0.805), a relationship of significance was observed regarding participants’ understanding of tools and resources and sector of employment, indicating 100% of responses that disagreed on having a good understanding of tools and resources fell within the private sector.

### Audiological rehabilitative training

Audiologists play an integral role during the counselling process, which highlights the importance of ensuring that audiologists are well equipped with the appropriate training in audiological rehabilitation (Meibos, Muñoz, & Twohig, 2019). Presently, there are five tertiary institutions within South Africa offering the degree at undergraduate and postgraduate levels. Results in this study revealed that of the 41 participants (n = 41), 68.3% of the participants (*n* = 28) obtained their degrees in KZN, whilst 29.3% (*n* = 12) obtained their degrees outside of KZN and 2.4% (*n* = 1) obtained their degrees from both KZN and outside the province. Of all respondents, 72.5% (*n* = 29) participants reported that their institution provided them with training for both informational and adjustment counselling, whilst 22.5% (*n* = 9) reported only being offered training in informational counselling and 5% (*n* = 2) reported a lack of training in counselling. A significant relationship was observed between participants who felt they could have been better trained for counselling by their respective institutions and sectors of employment, with a *p*-value = 0.044, suggesting public sector audiologists presenting views of the need for improved training in counselling.

### Multicultural sensitivity

The American Speech Hearing Association (2012) advocated for the education of audiologists to ensure their capacity for multicultural sensitivity during counselling practices.

Most participants 75.6% (*n* = 31) reported accommodating a multicultural population whilst 24.4% (*n* = 10), reported not accommodating for multicultural sensitivity. There were 29 responses explaining how multicultural sensitivity was adhered to or not adhered to during practice, a majority of 17 respondents reported that adhereing to multicultural sensitivity was attributed to inclusion of bilinguism during practice, while 3 respondents attributed not adhering to multicultural sensitivity to language barriers and tools which did not fit the context of the population managed. Whilst 50% (*n* = 20) of participants agreed on tools used for presbycusis and tinnitus counselling being multiculturally sensitive, 12.5% (*n* = 5) of participants disagreed and a further 7.5% (*n* = 3) strongly disagreed. There were no significant relationships established around multicultural sensitivity and sectors of employment and years of experience.

### Patient satisfaction

Audiologists with essential skills conducive to counselling increase patient satisfaction. Audiologists reported on their ability to ensure patient satisfaction through counselling practices (De Man et al., 2016).

All 41 participants conducted a satisfaction survey based on how they perceived their patients to be satisfied with their services. Most audiologists *n* = 33 (80%) agreed that their counselling practices provided benefits to their patients. There was no significant relationship between audiologists viewing their patients’ benefits from counselling practices and years of experience (*p* = 0.753) or sectors of employment (*p* = 0.915). Furthermore, 39 out of 41 audiologists agreed that the patient’s perceptions could provide input on improving counselling practices.

## Discussion

**FIGURE 2:**
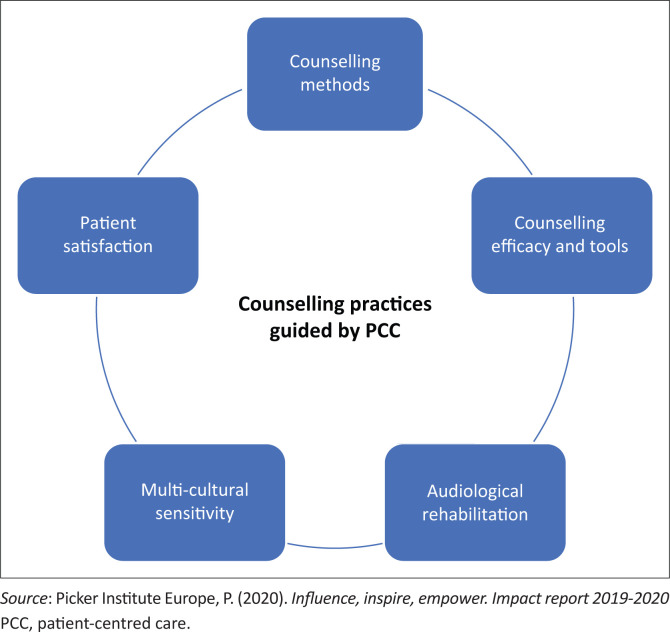
Adapted framework on implementing patient-centred care into counselling practices.

Patient-centred care can be implemented into audiology practice by focusing on PCC dimensions suitable for counselling, these being the Picker principles mentioned earlier (Picker Institute Europe, 2020). Literature indicates select dimensions of PCC being related to the specific context of services provided (Berghout, Van Exel, Leensvaart, & Cramm, 2015), those services being counselling in this study. Furthermore, there is a need for conceptualising PCC implementation in current audiology practices (Grenness, Hickson, Laplante-Lévesque, & Davidson, 2014). The following five components of counselling practice were focused on during this study: counselling methods, counselling efficacy and tools, audiological rehabilitative training, multicultural sensitivity and patient satisfaction, as depicted in Figure 2.

The researchers adapted the Picker principles to form the framework focusing on implementing PCC within audiology counselling practices. This assisted in understanding how a practice can be investigated using these five constructs.

### Counselling methods

Counselling methods assist in informing and educating patients. This can be conducted in various ways that involve information, communication and education, which are viewed as the most important dimensions of PCC (Berghout et al., 2015). Counselling methods utilised by audiologists can thus serve as a medium for implementing PPC dimensions into practice.

Common management of presbycusis is compensating for the hearing loss with assistive hearing devices, placing little attention on counselling (Parham et al., 2013). Management for tinnitus include pharmacological drug administration typical in developed countries and non-pharmacological forms of treatment that include counselling (Han et al., 2009). Results from this study revealed that a majority of audiologists (82.9%) managed presbycusis and tinnitus using both methods, compensating for the hearing loss with a hearing device and providing counselling services. A minority provided only compensation for hearing loss through an assistive hearing device, which is still laudable; however, a shift towards a combination of compensation and counselling is required to address the psychosocial aspects of presbycusis and associated tinnitus for improved quality of life (Parham et al., 2013). The study related the findings of audiologists concentrating more on compensating for the hearing loss; whilst this may benefit patients, it does not adequately address all aspects of quality of life (Parham et al., 2013). Implementing PCC into presbycusis and associated tinnitus counselling practices should therefore be a major aspect of treatment as this would ensure holistic management for patients, as previously advocated in the literature. Counselling is thus a necessity during the management process and should be provided through appropriate methods.

Appropriate counselling methods used by audiologists include informational and adjustment counselling (American Speech Hearing Association, 2012). Both methods provide for information-sharing, patient education, respect for patient preferences, emotional support of patients and inclusion of the patient’s significant others, all of which are aspects of the eight Picker principles (Picker Institute Europe, 2020) falling within the context of PCC. The American Speech Hearing Association (2012) describes informational counselling as a form of teaching, in which the audiologist imparts knowledge related to the hearing diagnosis to the patient and significant others, whereas adjustment counselling explores the experiences of the patient, identifying the conflicts and emotions they go through (British Society of Audiology, 2016) to deal with the diagnosis. Whilst a majority of audiologists (75.6%) offered both informational and adjustment counselling, the minority (24.4%) of audiologists solely provided informational counselling. It was further revealed that there was no significant relationship between years of experience and informational and adjustment counselling. This supports previous research that has shown audiologists are more inclined towards informational counselling (Meibos et al., 2017); this study suggests that this inclination is irrespective of the audiologists’ years of experience. The inclination in favouring informational counselling may be attributed to higher confidence levels amongst audiologists, obtained through training, and increased knowledge on informational counselling as opposed to adjustment counselling (Meibos et al., 2017). This insignificant relationship between years of experience and informational and adjustment counselling may further indicate that currently audiologists are not receiving the necessary training required to improve their confidence levels in adjustment counselling. This is substantiated by a study conducted in South Africa that found audiologists showed difficulty in facilitating adjustment counselling and attributed it to a lack of knowledge, training and confidence (Makhoba & Joseph, 2016). This is further perceived by patients who are discussed at length in a subsequent article, exploring patients’ perceptions of counselling practices. Similarly, this study speculates that the preference towards informational counselling may be attributed to a lack of training and skill in adjustment counselling, indicating the need for further training of already practising audiologists or improved training for undergraduate audiology students. To support the recommendation for increased and improved counselling training, an exploratory study conducted by Meibos et al. (2017) focused on training in counselling. Findings revealed improvement in counselling practices post-training and concluded that further training is needed for audiologists.

Apart from informational and adjustment counselling, this study reported on methods of counselling through individuals, families or groups (American Speech Hearing Association, 2012). Patient-centred care is characterised by the inclusion of the patient’s family and friends in shared decision-making and involvement (Smith, Saunders, Stuckhardt, & McGinnis, 2013). Delivery of these various options for counselling therefore ensures the implementation of PCC in practice. It was found that more than half of audiologists offered individual counselling (87.8%), followed by those who offered family counselling (53.7%), and the least number of audiologists offered group counselling (2.4%). Literature indicates a preference for group counselling but cautions that it should be dictated by patients’ context, resources and cultural practices (Meibos et al., 2019). However, this study indicated that audiologists showed an inclination towards individual (87.8%) and family (53.7%) counselling as compared with group counselling (2.4%). This was observed in both the private and public sectors. This was mainly attributed to the COVID-19 pandemic and patient preferences that were listed as contributing factors to inclination towards individual and family counselling over group counselling. This is supported by studies based on the South African healthcare system, revealing various disadvantages within the South African context, notably resource and time constraints and high patient loads (Maphumulo & Bhengu, 2019). Therefore, the preferences of the patient must be considered. Counselling methods can thus better adhere to PCC dimensions, ultimately improving other aspects of counselling practices, which include efficacy and tools.

### Counselling efficacy and tools

Efficacy of counselling describes the benefits or helpfulness of counselling practices. In assessing the efficacy of presbycusis and tinnitus counselling practices, audiologists must use appropriate measurement scales (British Society of Audiology, 2016). This study found that a majority of audiologists commonly used measurement scales during counselling practices, such as the Abbreviated Profile of Hearing Aid Benefit, THI and SCHHSE (British Society of Audiology, 2016). Literature suggests that these tools used during counselling practices assist in determining the patient’s overall progress towards goals of improved quality of life and implement integrated care amongst audiologists (British Society of Audiology, 2016). However, measurement scales such as the Client-Oriented Scale of Improvement, Communication Scale for Older Adults and the WHO Disability Assessment Schedule II were not found to be used in practice, for unknown reasons. Supporting studies reporting audiologists not monitoring their counselling efficacy or appropriateness with the use of measurement scales (De Man et al., 2016) therefore prove to be evident in current practice. This was further corroborated in the subsequent study focusing on patients’ perceptions of tools and resources. However, a minority of audiologists did not find tools and resources to be helpful during counselling practice, which they attributed to context specificity and language barriers. This may be the main contributing factor as to why certain measurement scales are not included in practice. Furthermore, it may have a negative impact on services, as contrary literature suggests that these tools have proved to be internationally efficient in measuring counselling efficacy and providing guidance to audiologists regarding counselling practices (American Speech Hearing Association, 2012; British Society of Audiology, 2016). Thus, there are measurement scales that serve as tools for counselling and may be used as a guideline to counselling practices that improve efficacy and patient outcome. This study revealed that there are tools that are not being used in current practice, even though they are widely available. This may be attributed to availability of these tools to audiologists and context-specificity of tools and resources; audiologists reported that tools were not context-specific in terms of linguistic significance, which impacts their use. Audiologists with 5–10 years of experience felt that the use of tools and resources showed no significant impact in ensuring the efficacy of counselling practices; however, these tools have been documented to focus on the patient’s individual needs and improve patient satisfaction (British Society of Audiology, 2016), complementing the dimensions of PCC. Therefore, this study suggests that audiologists with 5–10 years of experience who participated in this study might be underestimating the impact of tools and resources on counselling efficacy, and this may be a contributing factor to the disuse of these tools and resources. It was further discovered that audiologists have been including resources during counselling practices such as pamphlets that may remain with the patient. Such practice is in line with the PCC dimension of patient information and education, as sharing written information that has been proven to assist patients with hearing loss is admirable (Balachandran, 2020). Therefore, whilst measurement tools and resources suggested for counselling practices are advantageous internationally, there are still limitations that are encountered through context specificity. This may be improved by refining tools and resources to fit the context and improve the training of audiologists in implementing such tools to improve outcomes.

### Audiological rehabilitative training

Meibos et al. (2019), found that an audiologist’s competency in counselling acquired through training contributes to counselling efficacy. Investigating audiological rehabilitative training was thus crucial in gaining knowledge about audiologist training in counselling practices. The study found that whilst a majority of audiologists reported having been trained in both informational and adjustment counselling, a minority of audiologists reported having been trained only in informational counselling or to have received no training in counselling at all. This is related to the inclination towards informational counselling over other options of counselling delivery, which has been noticed in the past research based in South Africa (Makhoba & Joseph, 2016). This was further corroborated by patients’ perceiving and recommending improved adjustment counselling to be included in practice, which is focused on in the subsequent study. This further indicates that in a span of 5 years, audiologists within a similar context may still have limitations in skill development because of institutional training that may impact providing emotional support to patients, thereby negatively impacting PCC dimensions. Furthermore, audiologists who reported on not receiving any training in either informational or adjustment counselling reported not having a good understanding of the use of tools and resources during counselling, indicating that audiologists’ lack of skills training may strain their ability to efficiently counsel patients. This is supported by literature revealing that audiologists’ skills or training in counselling impact their preparedness to counsel (Meibos et al., 2017). These sentiments add to the notion that to provide appropriate counselling practices, audiologists need to be trained to reach a point of preparedness that allows them to counsel patients using PCC.

This study further found that that there is a significant relationship of *p* = 0.044 between audiologists employed in the public sector reporting an increased need for improved training in counselling as compared with audiologists in the private sector. This can be attributed to differing requirements of counselling based on resource constraints and barriers between sectors. This is supported by studies specific to South Africa, indicating that audiologists tend to reflect differing practices based on their training and skills (Makhoba & Joseph, 2016). This may suggest that the training of audiologists may not be sufficient for public sector requirements. A common report that has recurred throughout the study is that language barriers impact counselling practices; this indicates the need to ensure that institutions provide context-specific training to better equip audiologists in overcoming certain factors faced in the public sector, as supported by Khoza-Shangase & Mophosho (2018), suggesting that context-specific training may impact service delivery. A study by Pillay and Kathard (2018) suggests that improvement in training is imperative in impacting service delivery. Apart from service delivery, the inclusion of context-specific training may improve audiologists’ multicultural sensitivity during counselling practices.

### Multicultural sensitivity

Healthcare providers indicated patient preferences and values to be the most important dimensions of PCC (Berghout et al., 2015). They further indicated that a focus on patients’ preferences and listening to their needs according to their values would improve shared decision-making and patient outcomes (Berghout et al., 2015), indicating the necessity of multicultural sensitivity in practice. Within the field of audiology, respecting patients’ preferences leads to the understanding of the patient’s individual needs that aid in the development of management to improve their quality of life (Balachandran, 2020). Therefore, patient preferences and values may be taken into consideration by placing a focus on multicultural sensitivity. This study found that the majority of audiologists reported demonstrating multicultural sensitivity during counselling practices; however, a minority of audiologists reported not adhering to such trends, which is mainly attributed to language barriers faced during the counselling process between audiologists and patients. This is consistent with research based in a similar context that reported the impact of language barriers affecting audiological services (Makhoba & Joseph, 2016), which is important as the Health Professionals Council of South Africa (Health Professions Council of South Africa) recommends that audiologists ensure appropriate management in a culturally and linguistically diverse South Africa (Health Professions Council of South Africa, 2019). Furthermore, counselling practices that does not provide for a multicultural population may not be fully adhering to PCC dimensions. Substantiated by research that focused on 271 psychologists registered in South Africa who explored multicultural counselling competencies, the themes derived from data analysis included competencies in understanding patients’ respect, tolerance and trust (Ngcobo & Edwards, 2008), suggesting that multicultural sensitivity can be implemented through various paths such as empathy, respect, tolerance and trust. This correlates with a study conducted by De Man et al. (2016) that explored the current state and barriers to the implementation of PCC in sub-Saharan Africa; participants described their satisfaction with culturally sensitive service delivery with reports of therapists’ understanding, respect and tolerance. This emphasises the relevance of patient preferences during counselling and the positive impacts on patient satisfaction. However, it is also critical for audiologists and patients to share the same definition of multicultural sensitivity; patients’ perceptions of multicultural sensitivity are therefore focused on in the subsequent study, further impacting patient satisfaction.

### Patient satisfaction

The majority of audiologists reported their counselling practices as beneficial to their patients. Audiologists’ sentiments are consistent with literature advocating the significance of counselling and PCC in patient satisfaction (De Man et al., 2016). Furthermore, the majority of audiologists reported positively on patients’ input in improving counselling practices. By encouraging and accepting patients’ input, audiologists will be adhering to PCC dimensions. Supporting these sentiments is a cross-sectional survey on healthcare delivery, conducted on 216 patients with a mean age of 47–94, revealing that PCC showed positive patient satisfaction (Kuipers, Cramm, & Nieboer, 2019). This study highlights and strengthens the implementation of PCC into counselling practices that will improve patient satisfaction. This study supports the assertion that counselling should incorporate a component of patient satisfaction evaluation to improve the implementation of PCC within the South African (SA) context.

## Study’s limitation and strengths

This quantitative study included some open questions to get further insight into counselling practices that allowed for a holistic view. Furthermore, there was an inclusion of public and private sectors that provided a balanced view of the study, using PCC to guide the evaluation of responses, providing for a neutral standard. However, the study sample is limited to KZN; therefore, the generalisation of findings is only for KZN.

## Conclusion

This study has found that audiologists have been implementing PCC into current counselling practices as discussed here in terms of the Picker principle; however, they are not fully adhering to all dimensions of PCC in those aspects of counselling practices excerpted as counselling methods, counselling efficacy and tools, audiological rehabilitative training, multicultural sensitivity and patient satisfaction. Therefore, current counselling practises show room for improvement. Within counselling methods, audiologists have been adhering to PCC given their resource constraints, those mainly being language barriers and the COVID-19 pandemic. However, there is room to better implement PCC through adhering to education, information and patient preferences. The insight into counselling efficacy and tools indicates that although audiologists have reported the use of tools and resources, it has not been fully adhered to as literature suggests, which is mainly attributed to language barriers and context specificity. Audiologists further reported on the need to improve training to better prepare themselves for counselling patients, supporting literature that advocates similar sentiments (Makhoba & Joseph, 2016). With improved training, audiologists can expect improved skills to better prepare themselves in ensuring multicultural sensitivity when in conjunction with other above-mentioned aspects of counselling practices, thus improving patient satisfaction. Therefore, patient satisfaction may be improved by the implementation of PCC in practice, specific to the context of services (Berghout et al., 2015). Whilst the conceptual framework depicted in this study will aid in the evaluation and implementation of PCC into practice, further research into the implementation and effectiveness of PCC in audiology practices within the KZN context is crucial for the field to move closer to adhering to PCC. There is a lack of evidence-based research on the relationship between PCC and patient outcomes (Rathert, Wyrwich, & Boren, 2013), which contributes to the knowledge gap in South Africa on PCC implementation in audiology practice. This necessitates the need for further research within this field of study.
